# Supplementary material for: Ligand effect on switching the rate-determining step of water oxidation in atomically precise metal nanoclusters
Source: Nat Commun. 2023 Jun 8;14:3374. doi: 10.1038/s41467-023-38914-7 (PMC10250400; doi:10.1038/s41467-023-38914-7)
Supplement: Supplementary file 1 — Supplementary Information [file 41467_2023_38914_MOESM1_ESM.pdf]

Supplementary Information

Ligand Effect on Switching the Rate-Determining Step of Water Oxidation in Atomically Precise Metal Nanoclusters

Zhihe Liu,[1, 2] Hua Tan,[3] Bo Li[4] Zehua Hu,[3] De-en Jiang[4] Qiaofeng Yao[1]*, Lei Wang[2]* and Jianping Xie[1, 2]*

1 Joint School of National University of Singapore and Tianjin University
International Campus of Tianjin University Binhai New City
Fuzhou 350207, P. R. China

2 Department of Chemical and Biomolecular Engineering
National University of Singapore, 117585, Singapore

3 H. Tan, Z. Hu
Division of Physics and Applied Physics, School of Physical and Mathematical Sciences
Nanyang Technological University, 637371, Singapore

4 Prof. D.-e. Jiang, Dr. B. Li
Department of Chemical and Biomolecular Engineering, Vanderbilt University,
Nashville, TN, 37235, USA

Email: chexiej@nus.edu.sg; wanglei8@nus.edu.sg; qfyao@tjufz.org.cn

Table of Contents

Experimental details 3

Chemicals and Materials. 3

Computational formular 3

Supplementary Result and discussion 4

Supplementary Fig. 1 XPS spectra of S 2p in different ligands. 6

Supplementary Fig. 2 Optical images of obtained Au_{25} NCs aqueous solutions 7

Supplementary Fig. 3. TEM images of $[Au_{25}(pMBA)_{18}]^-$ NCs. 8

Supplementary Fig. 4 TEM images of $[Au_{25}(HCys)_{18}]^-$ NCs. 9

Supplementary Fig. 5 TEM images of $[Au_{25}(MHA)_{18}]^-$ NCs. 10

Supplementary Fig. 6 Optical images of H-type cell. 11

Supplementary Fig. 7 LSV curve comparison for $[Au_{25}(pMBA)_{18}]^-$ and Au-pMBA complex.. 12

Supplementary Fig. 8 TEM images of Au NPs capped by pMBA. 13

Supplementary Fig. 9 LSV curves of $[Au_{25}(MHA)_{18}]^-$ NCs and Au NPs in 1 M KOH. 14

Supplementary Fig. 10 Overpotentials comparison with the reference of 10 mA/cm^2. 15

Supplementary Fig. 11 CV curves of $[Au_{25}(MHA)_{18}]^-$ NCs in Faradic region. 16

Supplementary Fig. 12. CV curves of $[Au_{25}(HCys)_{18}]^-$ NCs in Faradic region.. 17

Supplementary Fig. 13 CV curves of $[Au_{25}(pMBA)_{18}]^-$ NCs in Faradic region.. 18

Supplementary Fig. 14 CV curve of $[Au_{25}(MHA)_{18}]^-$ in the non-Faradic region. 19

Supplementary Fig. 15 CV curve of $[Au_{25}(HCys)_{18}]^-$ in the non-Faradic region. 20

Supplementary Fig. 16 CV curve of $[Au_{25}(pMBA)_{18}]^-$ in the non-Faradic region. 21

Supplementary Fig. 17 Comparison of double layer capacitance value of Au_{25} NCs 22

Supplementary Fig. 18 Correlationship between Au(I)/Au(0) and TOF. 23

Supplementary Fig. 19 i-t curves for $[Au_{25}(HCys)_{18}]^-$ NCs. 24

Supplementary Fig. 20 i-t curves for $[Au_{25}(MHA)_{18}]^-$ NCs. 25

Supplementary Fig. 21 UV-vis spectra before and after homogenous OER for Au_{25} NCs capped by pMBA. 26

Supplementary Fig. 22 ESI-MS before and after homogenous OER for Au_{25} NCs capped by pMBA.. 27

Supplementary Fig. 23 UV-vis spectra before and after homogenous OER for Au_{25} NCs capped by HCys. 28

Supplementary Fig. 24 UV-vis spectra before and after homogenous OER for Au_{25} NCs capped by MHA 29

Supplementary Fig. 25 ESI-MS before and after homogenous OER for Au_{25} NCs capped by HCys. 30

Supplementary Fig. 26 ESI-MS before and after homogenous OER for Au_{25} NCs capped by MHA. 31

Supplementary Fig. 27 Optical images of confocal Raman microscope and *in situ* Raman electrolyser. 32

Supplementary Fig. 28 Calculated adsorption energy profiles of OH 33

Supplementary Fig. 29 Calculated adsorption energy profiles of O 34

Supplementary References 35

Experimental details

Chemicals and Materials: Ultrapure Millipore water (18.2 MΩ) was used throughout the experiments. Gold tetrachloride trihydrate ($HAuCl_4 \cdot 3H_2O$), *para*-mercaptobenzoic acid (pMBA), 6-mercaptohexanoic acid (6-MHA), Homocysteine (HCys), sodium hydroxide (NaOH), sodium borohydride ($NaBH_4$), N-Methyl-2-pyrrolidone (NMP) and N, N-dimethylformamide (DMF) were purchased from Sigma-Aldrich. Carbon monoxide (CO) with the purity of 99% without further purification was provided by *Singapore Oxygen Air Liquide Pte Ltd* (SOXAL). Ethanol was from Fisher. Carbon powder (Vulcan XC-72) and carbon fiber paper (Avcarb P75) was purchased by Fuel Cell Store. All chemicals were commercially available without further purification.

Computational formular

For all the electrochemical analysis, the working potentials versus Ag/AgCl were converted to a reversible hydrogen electrode (RHE) scale according to:

$$\mathrm{E_{RHE}} = \mathrm{E_{Ag/AgCl}} + 0.059 * pH + 0.198$$

The turnover frequency(s^{-1}) can be estimated from:

$$\mathrm{TOF} = I/(4\mathrm{F} * N)$$

where I is the current density ($A\ cm^{-2}$), can be calculated with $1cm^2$ working area for the different electrodes during the LSV measurement in 1.0 M KOH, F is the Faraday constant ($C\ mol^{-1}$), and N is the molar number of active sites for the different electrodes. The molar number of active sites is estimated by assuming all 12 Au atoms in the surface motifs of individual $[Au_{25}(SR)_{18}]^-$ are equivalent active sites. The concentration of $[Au_{25}(SR)_{18}]^-$ was measured by inductively coupled plasma optical emission spectroscopy (ICP-OES) on a Thermo Scientific iCAP 6000.

The Faradic efficiency reflects the utilization efficiency of electron in OER process. For OER (or HER) process, the Faradic efficiency can be obtained by calculating the ratio of the experimentally produced O_2 amount ($\boldsymbol{n_{O_2}}$) to the theoretical produced O_2 amount ($\boldsymbol{n_{O_2'}}$). Specifically, under a constant oxidation current (I) within a certain time (t), the experimentally produced O_2 amount can be measured by gas chromatography (GC). Thus, the Faradic efficiency can be calculated as following:

$$\text{Faradic Efficiency} = n_{O_2}/n_{O_2'} = 4\text{F} * n_{O_2}/I * t$$

The oxygen could be measured by a water gas displacing method in the constant potential-controlled test. The volume of O_2 were measured by gas chromatography and calculated from the following relationship:

$$V_{O_2} = Q * 22.4 * 1000/4\text{F}$$

where Q is the cumulative charge (C), F is the Faraday constant (C mol^{-1}).

The capacitive currents are measured in a potential range where no faradic processes are observed. We sweep the potential between 1.1~1.2 V vs RHE at different scan rates. The difference in current density variation ($\Delta j = j_a - j_c$) at the potential of 1.15 V vs RHE plotted against scan rate are fitted to estimate the electrochemical double-layer capacitances (C_{dl}).

Supplementary result and discussion

Quantized double layer (QDL) charging for monolayer protected metal NCs:

Quantized double layer capacitance charging had been observed for monolayer protected gold nanoclusters in the previous literatures.[1] Such capacitance results in the one-electron redox behaviors in metal clusters driven by the electric energy. The monolayer protected cluster can be regarded as a concentric sphere capacitor with the inner and outer spheres radii of r and r+d, (i.e., the core radius and the core radius plus thiolate monolayer thickness d). The thiolate monolayer exerts an effective dielectric constant ε. Therefore, the equation 1 for the concentric sphere capacitor is as follows:[2]

$$C_{CLU} = A_{CLU} \frac{\varepsilon\varepsilon_0}{r} \frac{r+d}{d} = 4\pi\, \varepsilon\varepsilon_0 \frac{r}{d}(r+d) \quad (1)$$

Where ε_0 and A_{CLU} is the permittivity of free space and core surface area, respectively. Assuming that the C_{CLU} is constant with core charge z to those core charge z-1, at the potential E_{app}, the ratio of core charge z to those with core charge z-t can be expressed if the cluster is placed on a macroscopic electrode:

$$\alpha_z = \frac{N_z}{N_{z-1}} = exp\left\{\frac{e}{k_B T}\left[E_{app} - E_{PZC} - \frac{(z-1/2)e}{C_{CLU}}\right]\right\} \quad (2)$$

E_{PZC} is the potential of zero charge for the nanoparticle core, Eq 2 with in Nernstian form demonstrates a QDL charging voltammetic wave shape should ideally be identical to that a reversible one-electron redox couple, with a formal potential characteristic of

the z/z-1 charge state change involved in metal NCs. The QDL behavior plays a key role for kinetics and mass transfer.

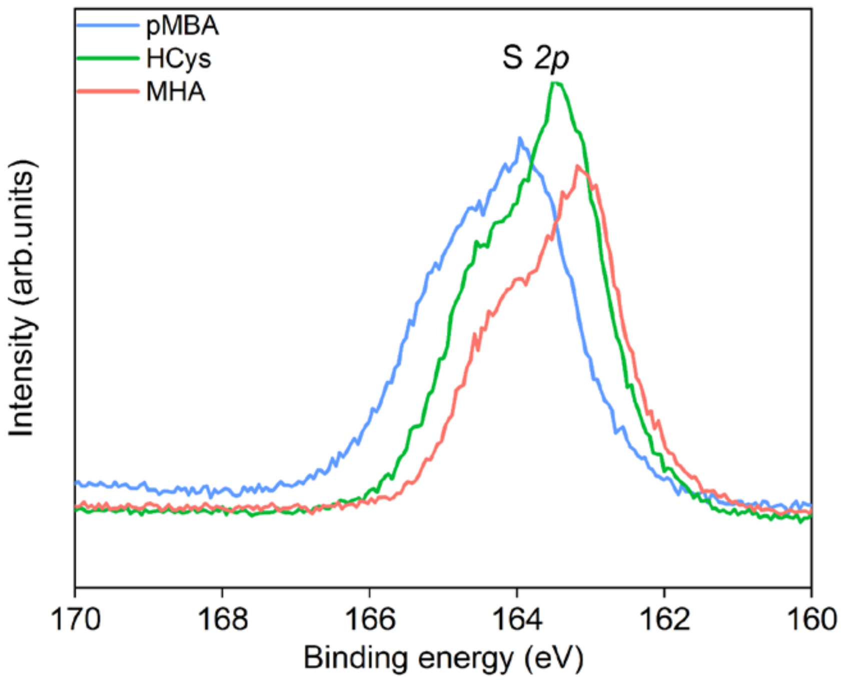

Supplementary Fig. 1 XPS spectra of S 2p in different ligands. The p-π electron delocalization between benzene ring and sulphur atom renders pMBA with stronger electron-withdrawing ability than the other two ligands. HCys have stronger electron-withdrawing ability than that of MHA, which was interpreted by the XPS spectra of S 2p. The positive shift of S 2p from MHA to HCys, to pMBA indicates the sequentially increasing electron-withdrawing capability of sulphur in ligands.

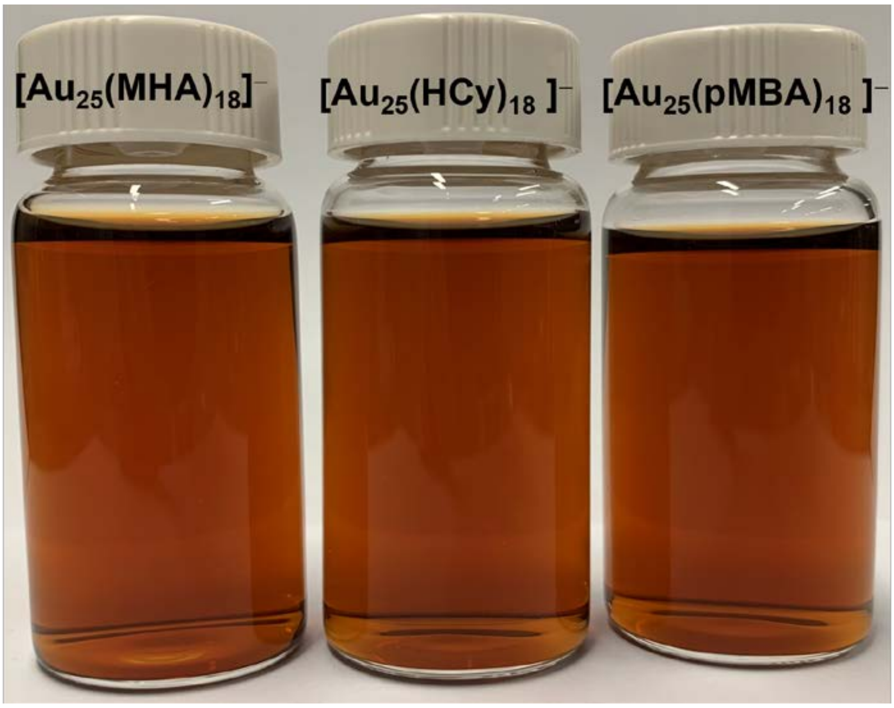

Supplementary Fig. 2 Optical images of obtained Au_{25} NCs aqueous solutions. The as-synthesized Au NCs are reddish brown in aqueous solutions.

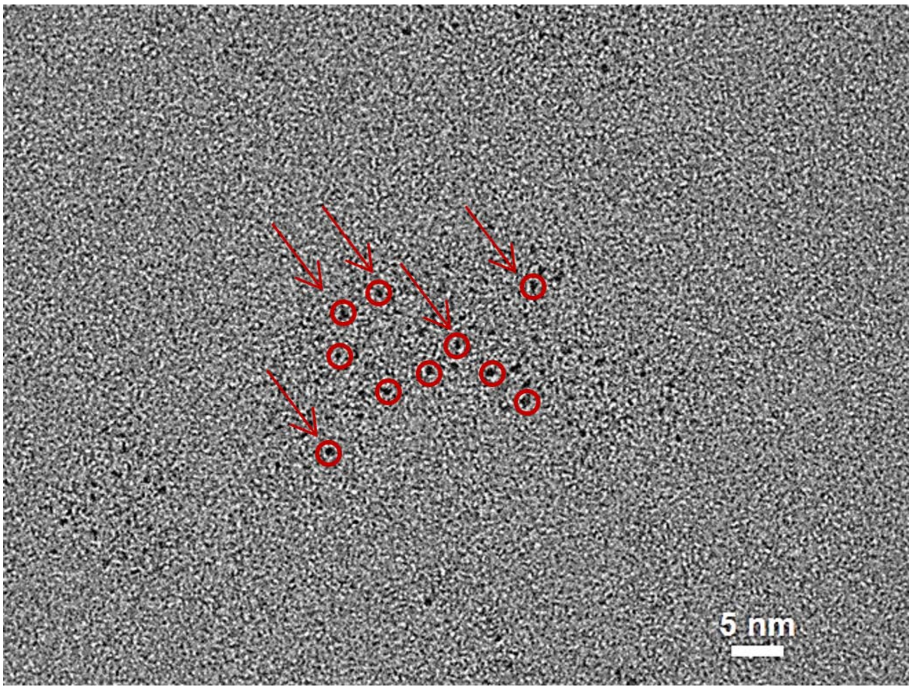

Supplementary Fig. 3. TEM images of $[Au_{25}(pMBA)_{18}]^{-}$ NCs.

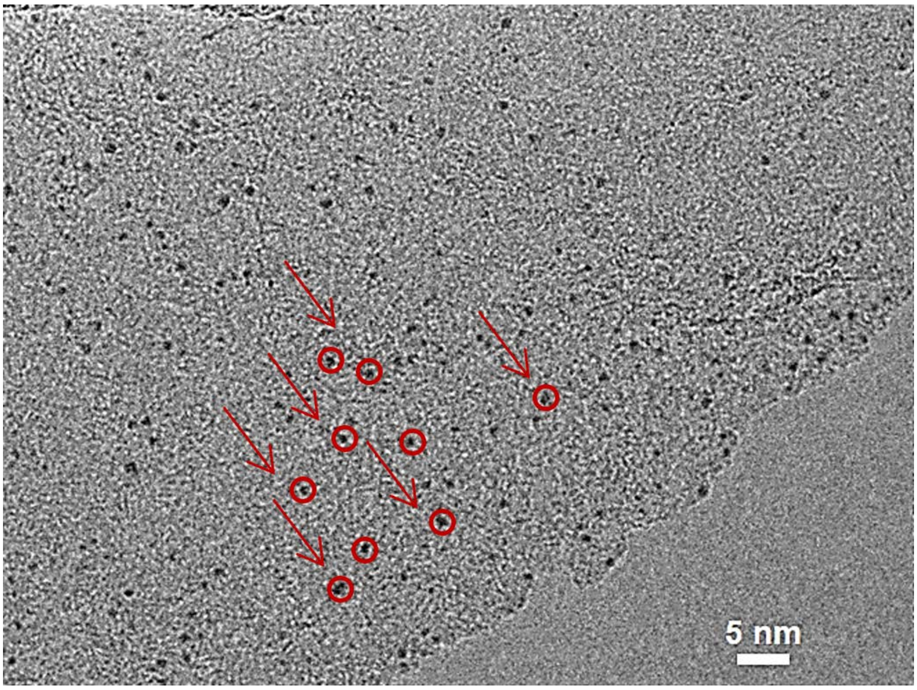

Supplementary Fig. 4 TEM images of $[Au_{25}(HCys)_{18}]^{-}$ NCs.

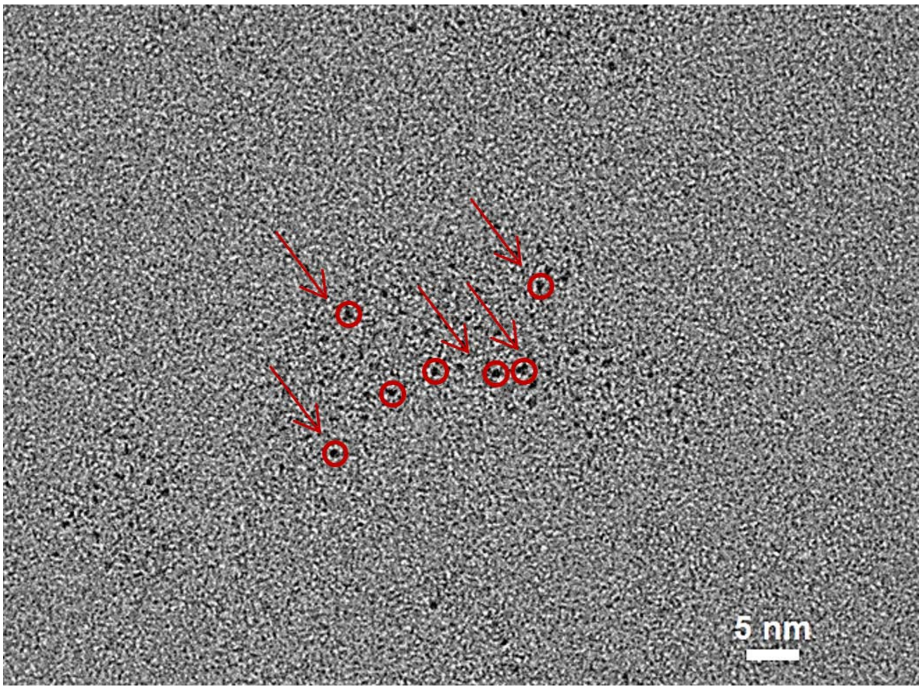

Supplementary Fig. 5 TEM images of $[Au_{25}(MHA)_{18}]^-$ NCs.

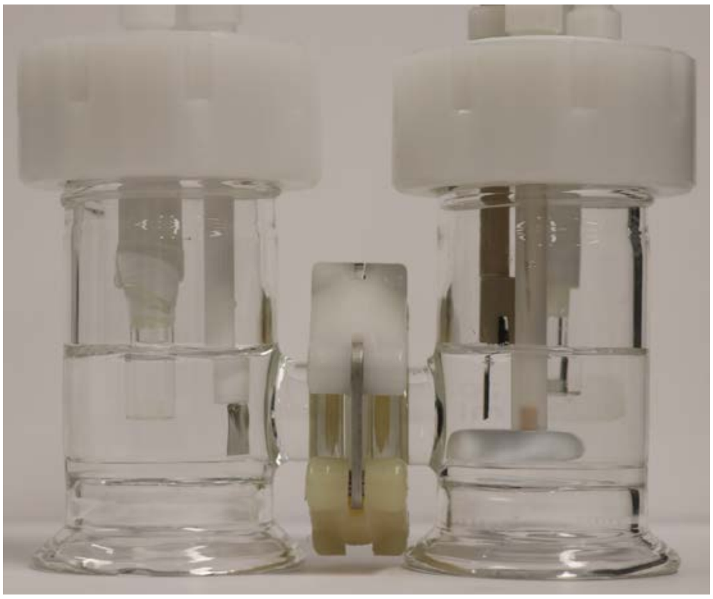

Supplementary Fig. 6 Optical images of H-type cell.

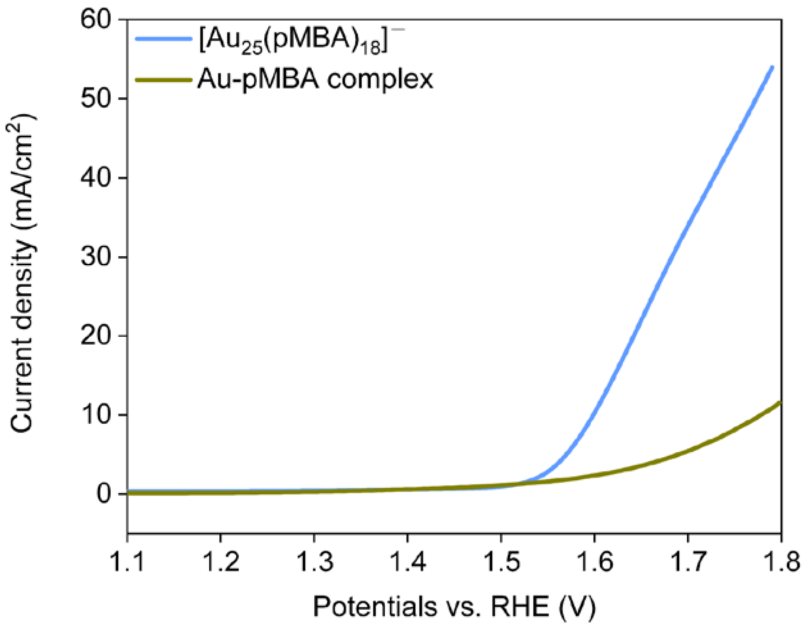

Supplementary Fig. 7 LSV curve comparison for $[Au_{25}(pMBA)_{18}]^{-}$ and Au-pMBA complex. The LSV curve comparison for $[Au_{25}(pMBA)_{18}]^{-}$ NC and Au-pMBA complex suggests the dominant role of cluster structure for effective electrocatalysis.

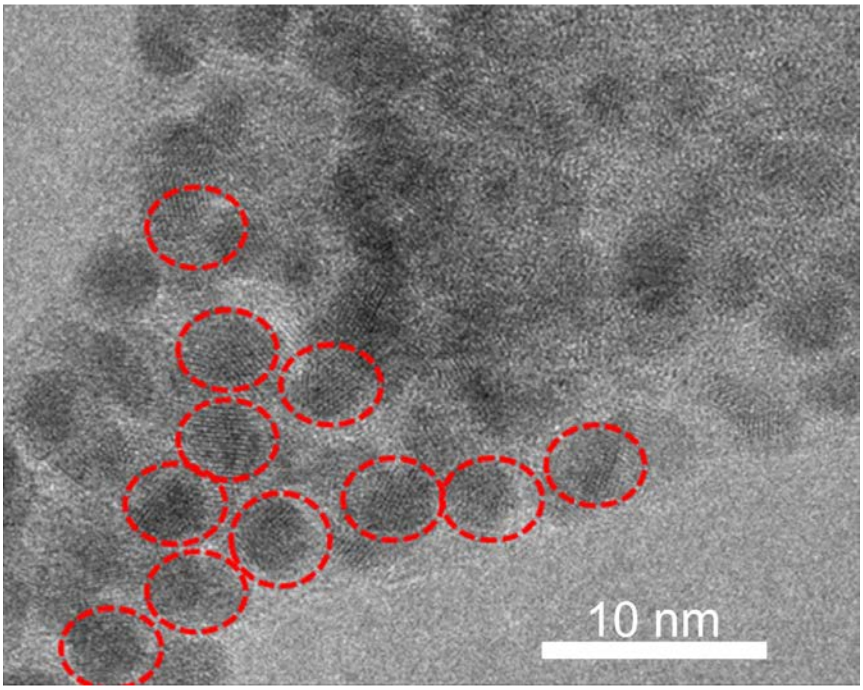

Supplementary Fig. 8 TEM images of Au NPs capped by pMBA.

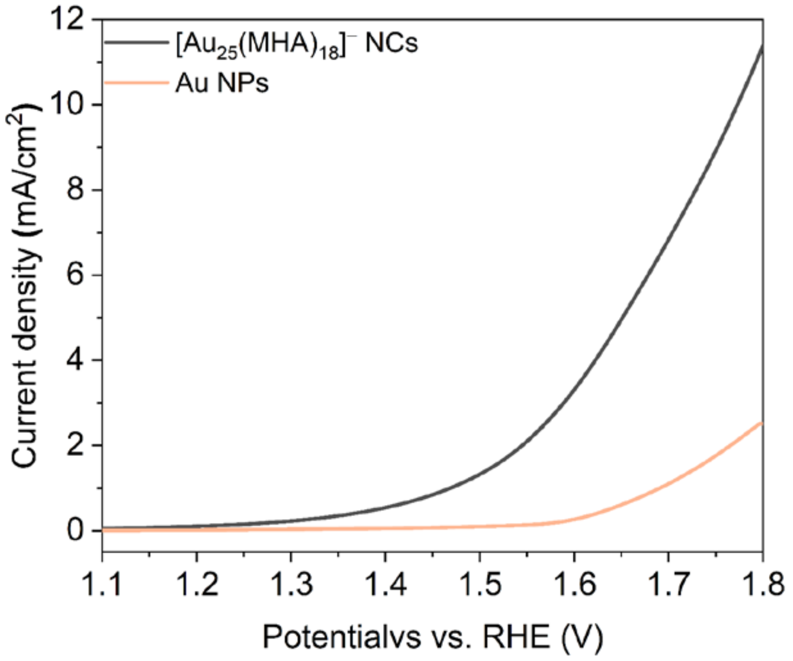

Supplementary Fig. 9 LSV curves of $[Au_{25}(MHA)_{18}]^{-}$ NCs and Au NPs in 1 M KOH. The LSV curves indicate that Au NPs deliver the highest current density of 2.5 mA/cm^2 within the same potential window from 1.1 to 1.8 V.

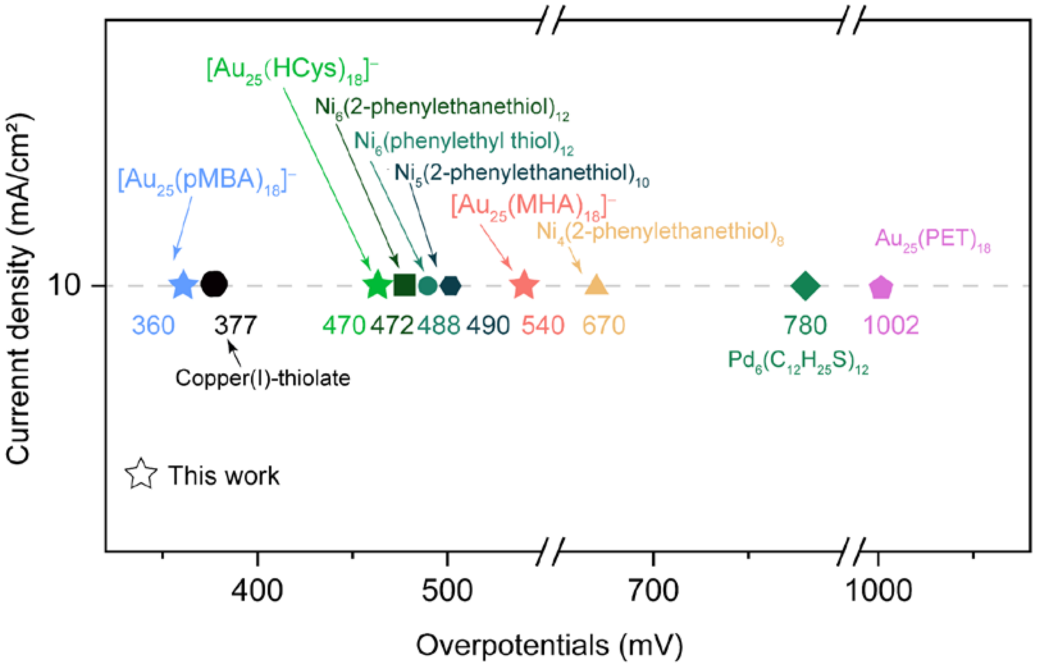

Supplementary Fig. 10 Overpotentials comparison with the reference of 10 mA/cm^2. (Ni_6(2-phenylethanethiol)$_{12}$, Ni_5(2-phenylethanethiol)$_{10}$, and Ni_4(2-phenylethanethiol)$_8$ (*Inorg. Chem.* 2023, 62, 1875−1884), $Pd_6(SC_{12}H_{26}S)_{12}$ (*Chem. Commun.* 2017, 53, 9733−9736), $Au_{25}PET_{18}$ (*Nanoscale* 2020, 12, 9969−9979), Copper (I)-thiolate cluster (*Chem. Commun.* 2020, 56, 3967−3970), Ni_6(phenylethyl)$_{12}$ (*ACS Catal.* 2016, 6, 1225−1234). The overpotentials in this work are marked in stars filled with different colors.

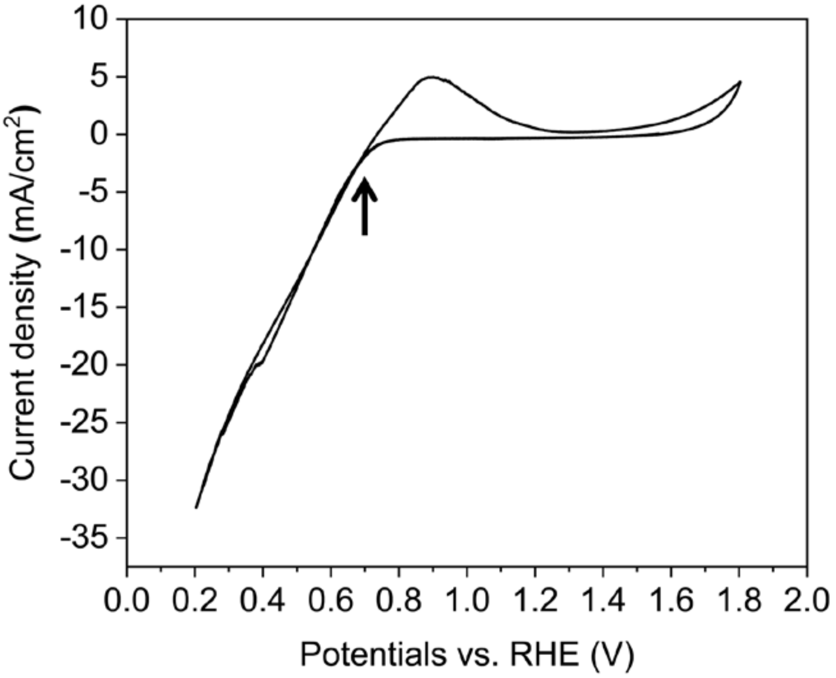

Supplementary Fig. 11 CV curves of $[Au_{25}(MHA)_{18}]^-$ NCs in Faradic region. The open-circuit potential position is marked as the arrow.

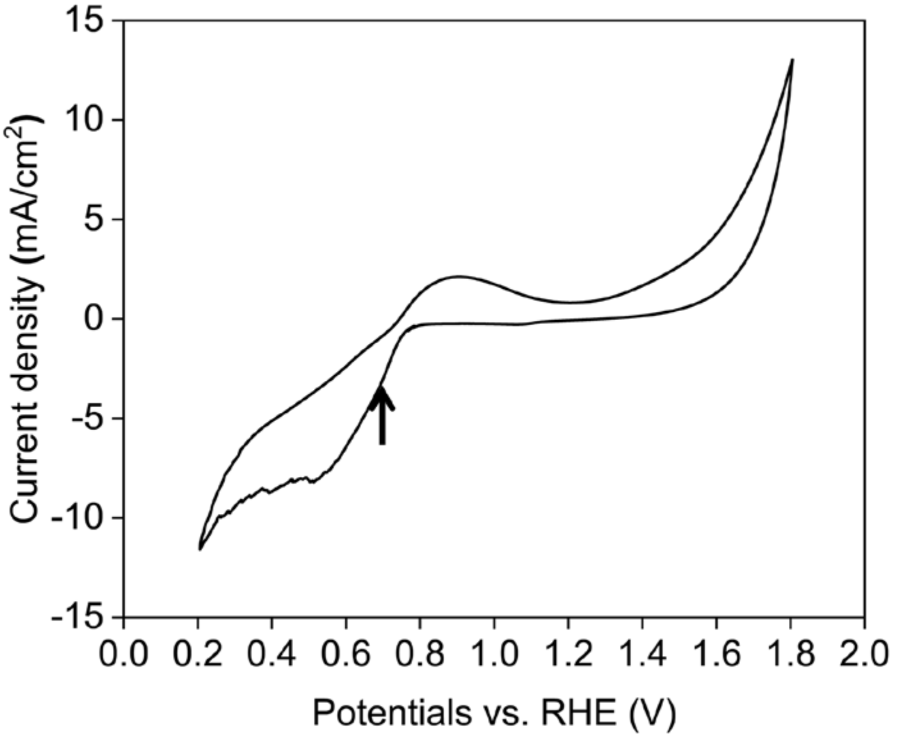

Supplementary Fig. 12. CV curves of $[Au_{25}(HCys)_{18}]^{-}$ NCs in Faradic region. The open-circuit potential position is marked as the arrow.

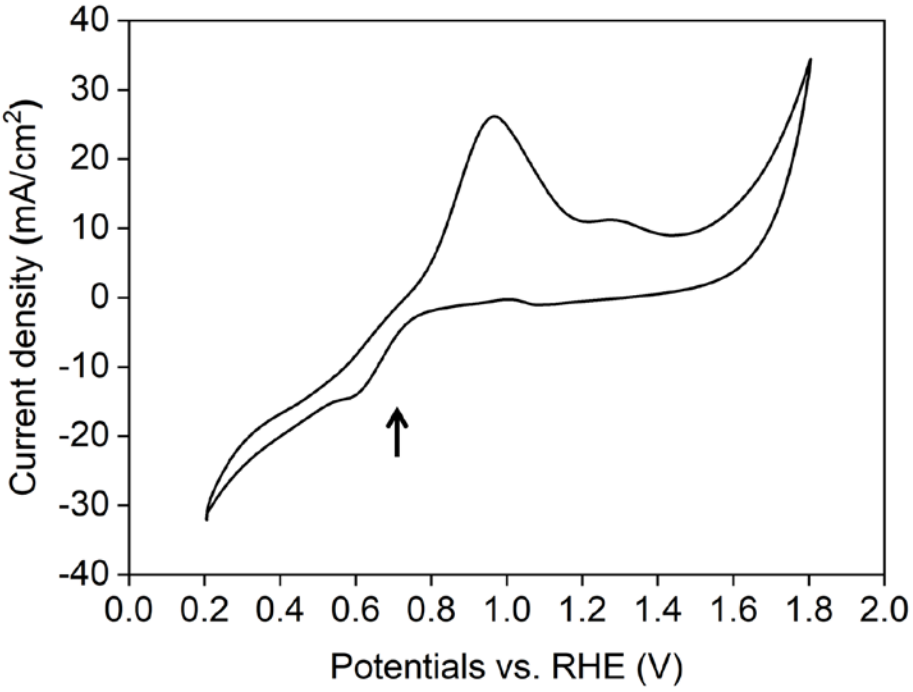

Supplementary Fig. 13 CV curves of $[Au_{25}(pMBA)_{18}]^{-}$ NCs in Faradic region. The open-circuit potential position is marked as the arrow.

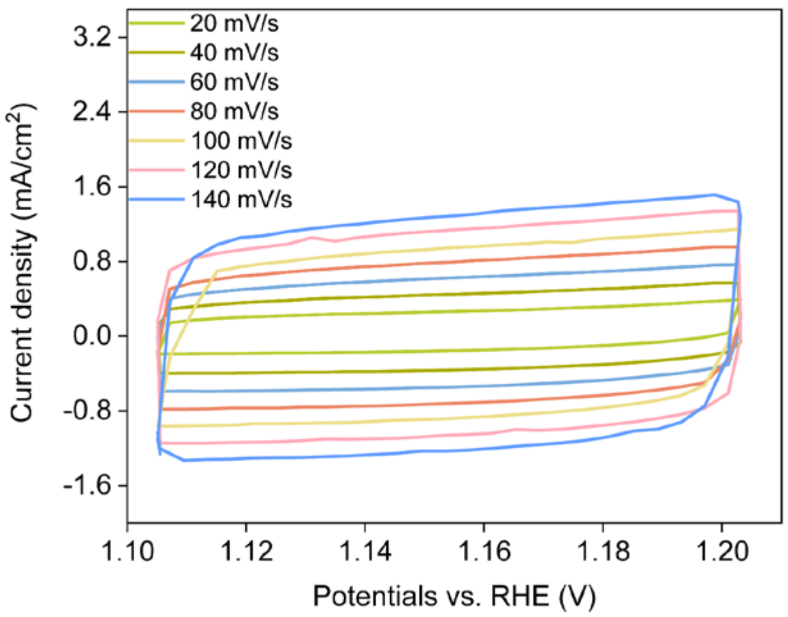

Supplementary Fig. 14 CV curve of $[Au_{25}(MHA)_{18}]^{-}$ in the non-Faradic region.

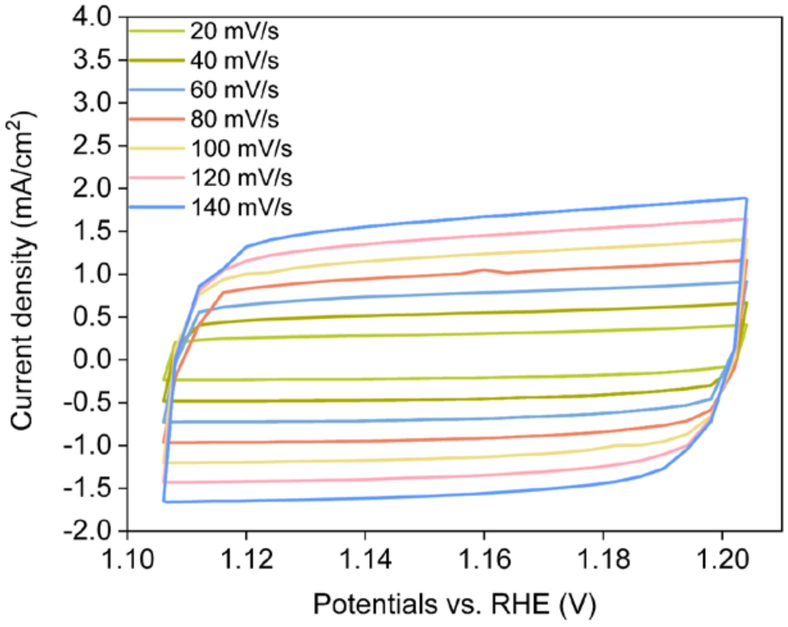

Supplementary Fig. 15 CV curve of $[Au_{25}(HCys)_{18}]^-$ in the non-Faradic region.

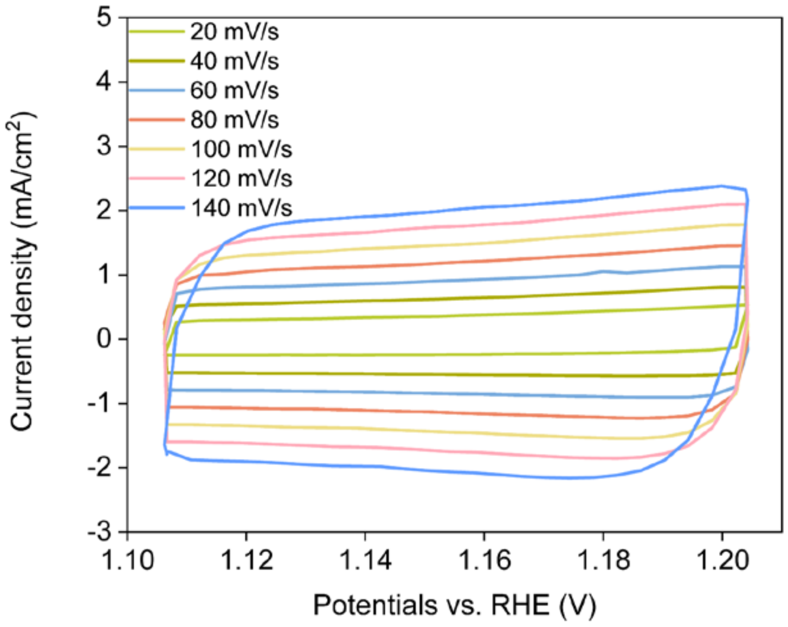

Supplementary Fig. 16 CV curve of $[Au_{25}(pMBA)_{18}]^-$ in the non-Faradic region.

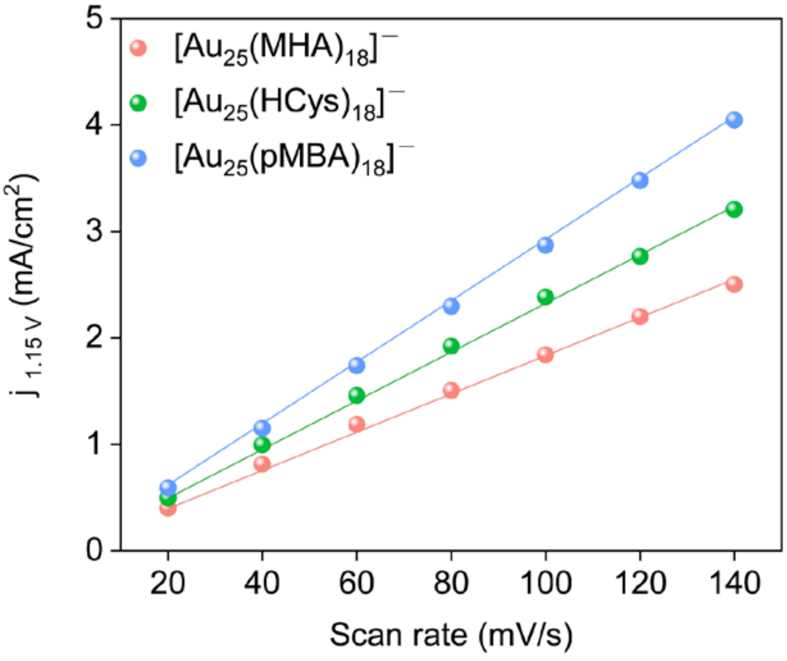

Supplementary Fig. 17 Comparison of double layer capacitance value of Au_{25} NCs capped by three different ligands.

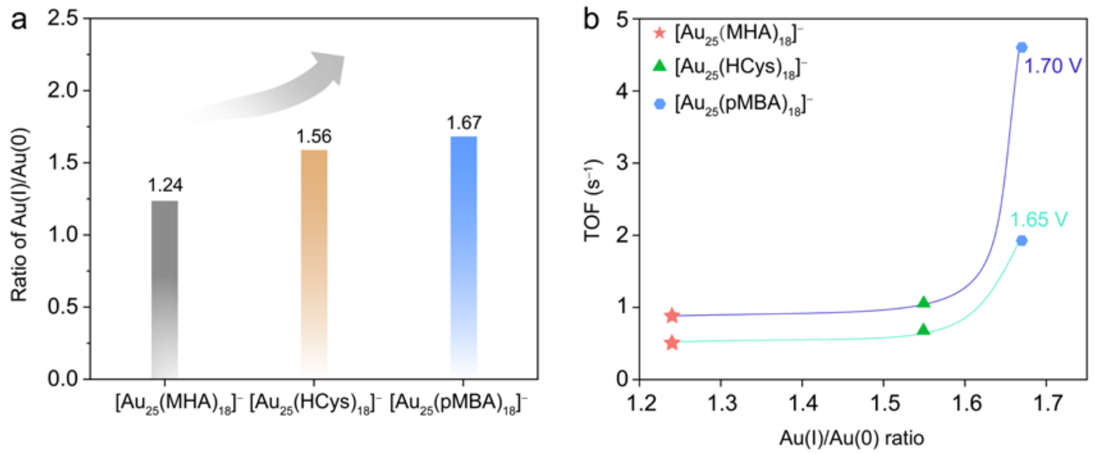

Supplementary Fig. 18 a Au(I)/Au(0) ratio for $[Au_{25}(SR)_{18}]^{-}$ NCs; b TOF vs Au(I)/Au(0) ratio for $[Au_{25}(SR)_{18}]^{-}$ NCs.

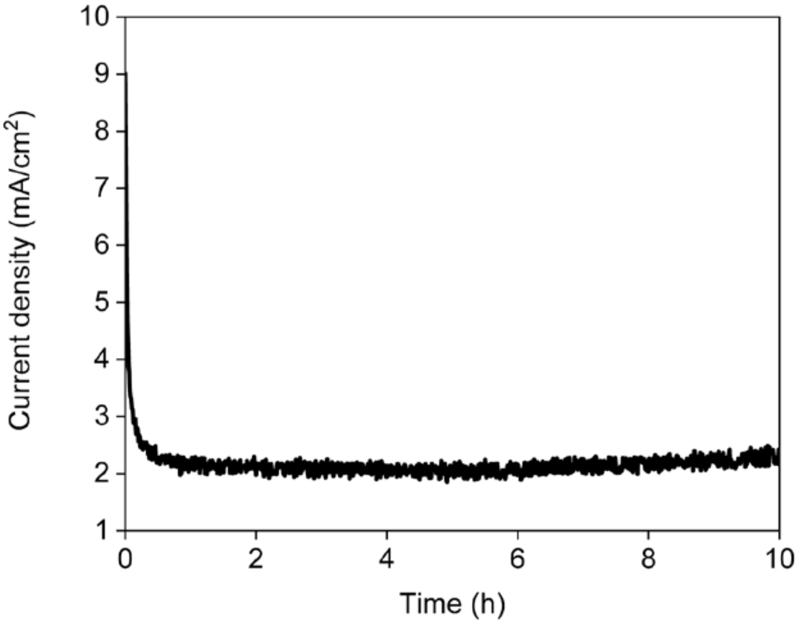

Supplementary Fig. 19 i-t curves for $[Au_{25}(HCys)_{18}]^-$ NCs.

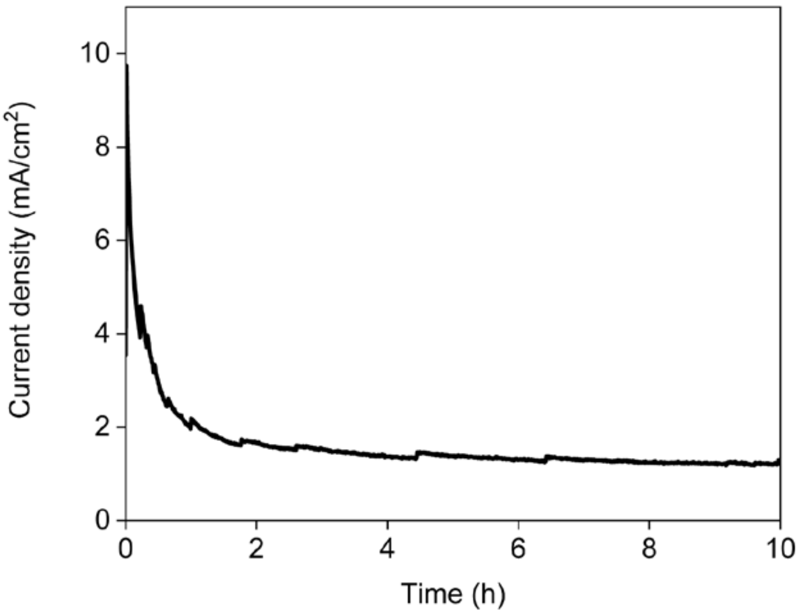

Supplementary Fig. 20 i-t curves for $[Au_{25}(MHA)_{18}]^-$ NCs.

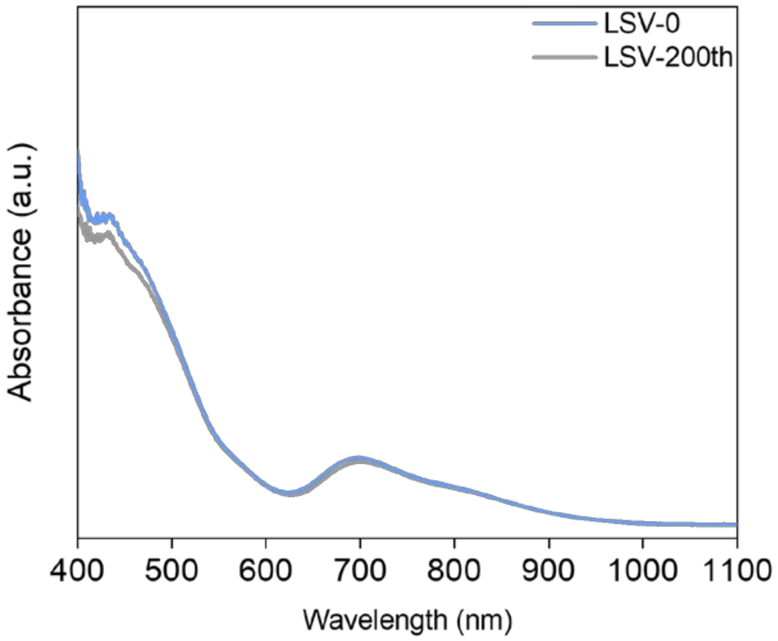

Supplementary Fig. 21 UV-vis spectra before and after homogenous OER for Au_{25} NCs capped by pMBA.

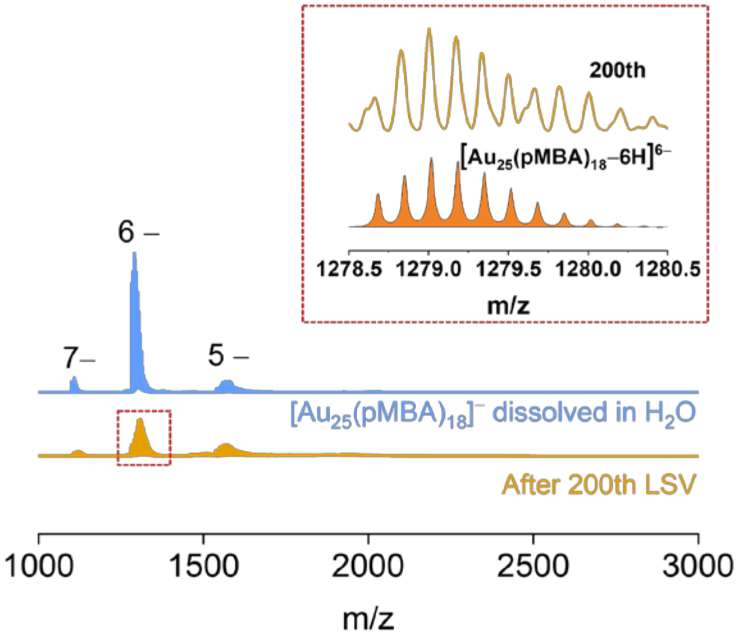

Supplementary Fig. 22 ESI-MS before and after homogenous OER for Au_{25} NCs capped by pMBA. Compared with the ESI-MS of $[Au_{25}(pMBA)_{18}]^{-}$ before OER, the ESI-MS for Au_{25} NCs capped pMBA after OER also carry 7, 6 and 5 negative charges, respectively. The experimental peak (unfilled solid line) shows the formula of Au_{25} NCs capped pMBA after 200 cyclic Linear sweep voltammetry (LSV) is $[Au_{25}(pMBA)_{18}\text{-}6H]^{0}$ with a reference to the simulated isotope patterns (insert of Fig. Supplementary Fig. 22). This reflects the Au_{25} capped by pMBA survive in the OER process.

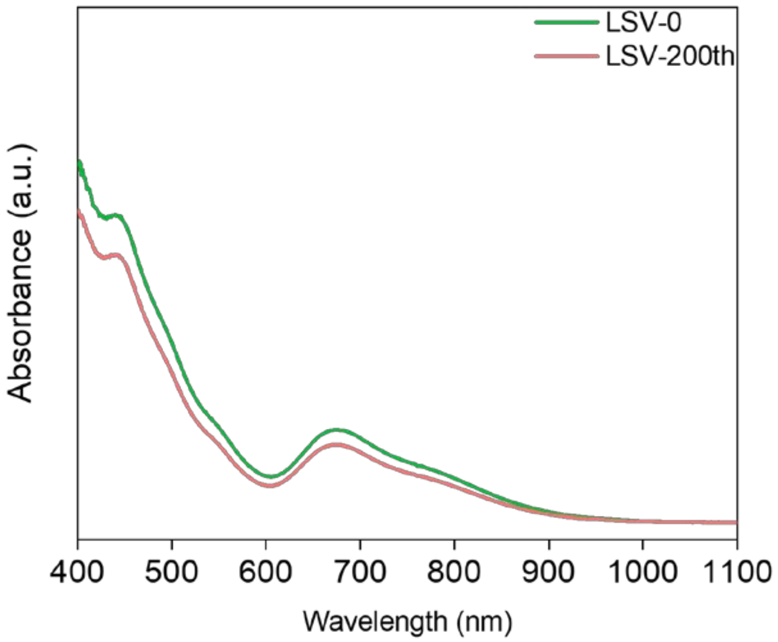

Supplementary Fig. 23 UV-vis spectra before and after homogenous OER for Au_{25} NCs capped by HCys.

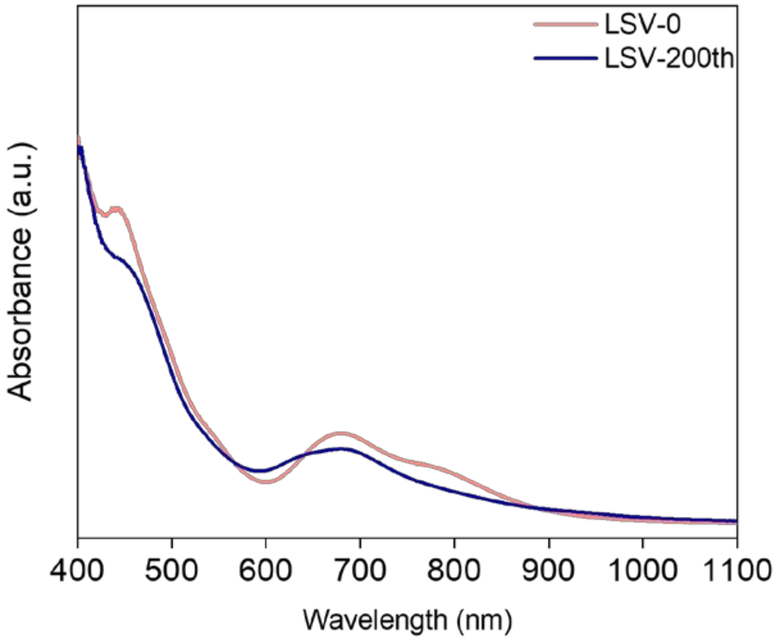

Supplementary Fig. 24 UV-vis spectra before and after homogenous OER for Au_{25} NCs capped by MHA.

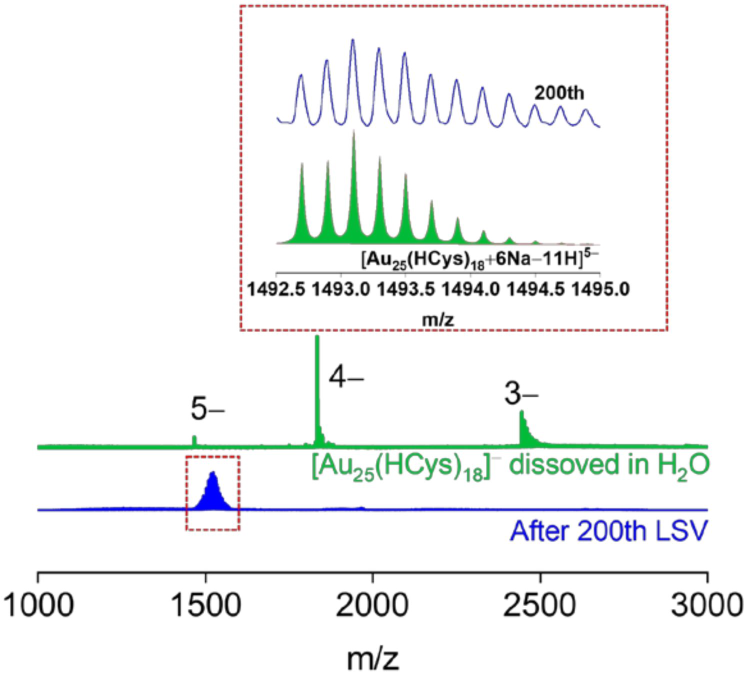

Supplementary Fig. 25 ESI-MS before and after homogenous OER for Au_{25} NCs capped by HCys. Compared with the ESI-MS of $[Au_{25}(HCys)_{18}]^{-}$ before OER, the ESI-MS for Au_{25} NCs capped HCys after OER also carry 5, 4 and 3 negative charges, respectively. The experimental peak (unfilled solid line) shows the formula of Au_{25} NCs capped HCys after 200 cyclic Linear sweep voltammetry (LSV) is $[Au_{25}(HCys)_{18}+6Na–11\ H]^{5-}$ with a reference to the simulated isotope patterns (insert of Fig. Supplementary Fig. 25). This reflects the Au_{25} NCs capped by HCys maintain the ligands in the OER process.

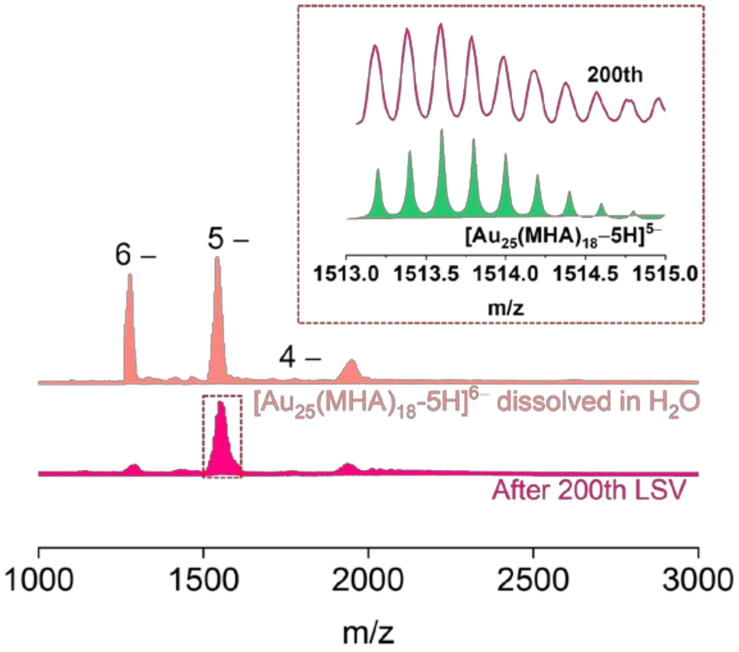

Supplementary Fig. 26 ESI-MS before and after homogenous OER for Au_{25} NCs capped by MHA. Compared with the ESI-MS of $[Au_{25}(MHA)_{18}]^{-}$ before OER, the ESI-MS for Au_{25} NCs capped MHA after OER also carry 6, 5 and 4 negative charges, respectively. The experimental peak (unfilled solid line) shows the formula of Au_{25} NCs capped MHA after 200 cyclic Linear sweep voltammetry (LSV) is $[Au_{25}(MHA)_{18}–5\ H]^{5–}$ with a reference to the simulated isotope patterns (insert of Fig. Supplementary Fig. 26). This reflects the Au_{25} NCs capped by MHA maintain the ligands in the OER process.

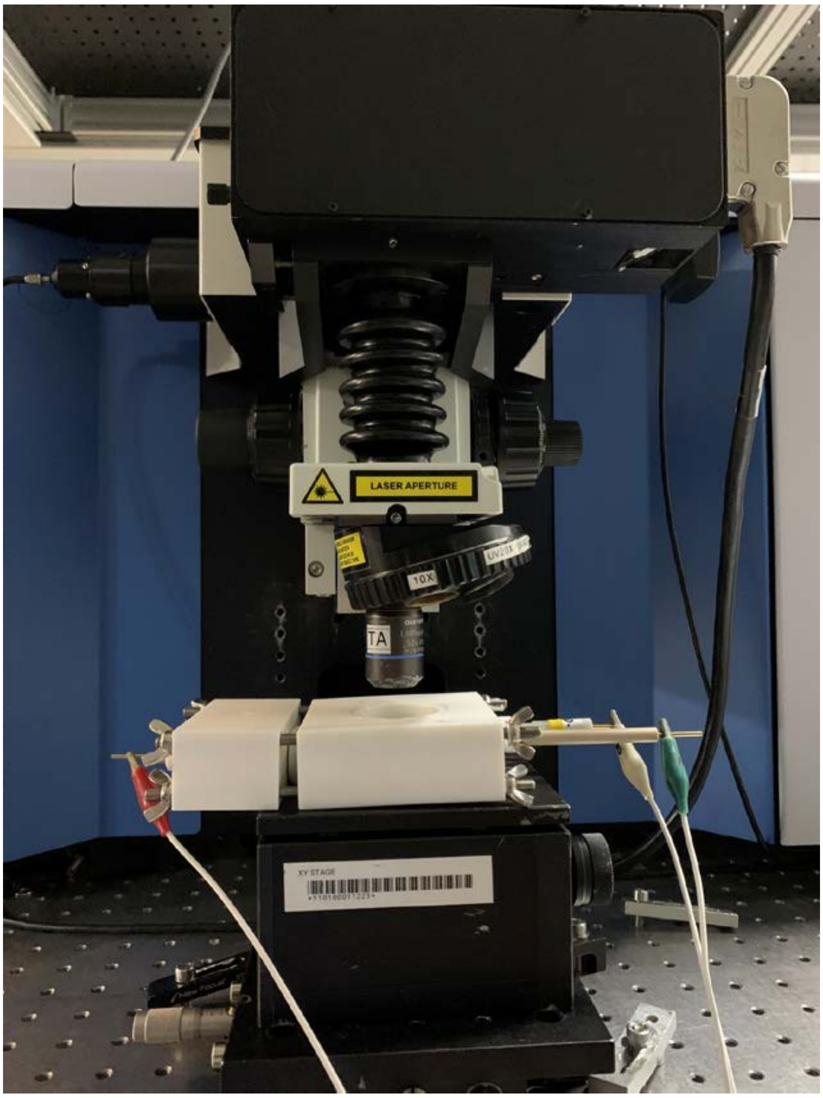

Supplementary Fig. 27 Optical images of confocal Raman microscope and *in situ* Raman electrolyser.

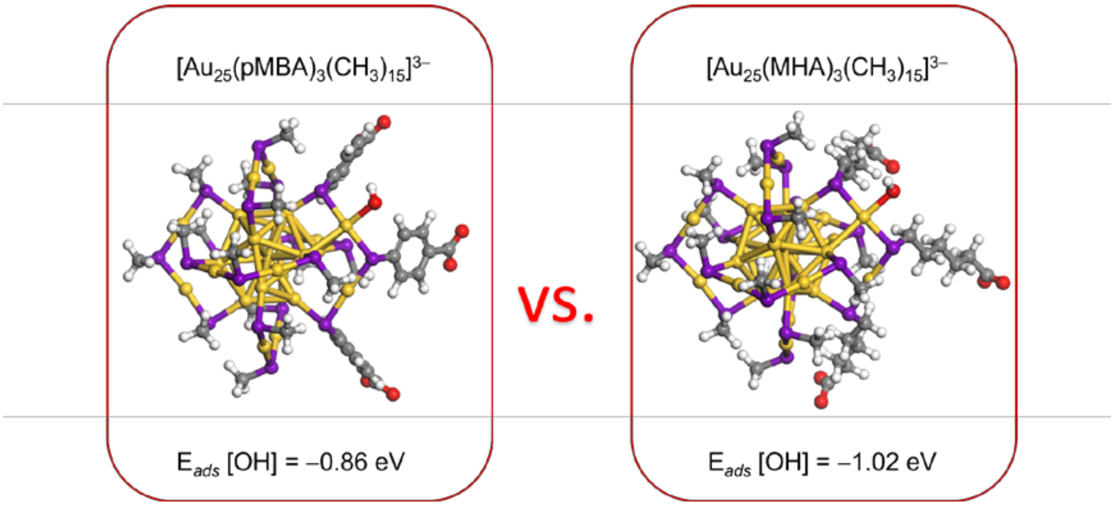

Supplementary Fig. 28 Calculated adsorption energy profiles of OH on Au_{25} NCs capped by pMBA and MHA.

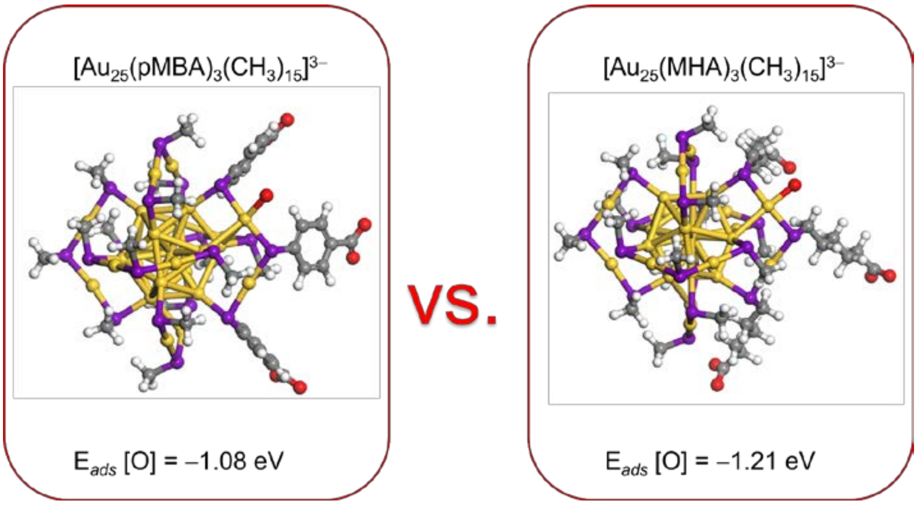

Supplementary Fig. 29 Calculated adsorption energy profiles of O on Au_{25} NCs capped by pMBA and MHA.

Supplementary References

1. Chen S, Higaki T, Ma H, Zhu M, Jin R, Wang G. Inhomogeneous Quantized Single-Electron Charging and Electrochemical–Optical Insights on Transition-Sized Atomically Precise Gold Nanoclusters. *ACS Nano* **14**, 16781-16790 (2020).

2. Hicks JF, Miles DT, Murray RW. Quantized Double-Layer Charging of Highly Monodisperse Metal Nanoparticles. *J. Am. Chem. Soc.* **124**, 13322-13328 (2002).
